# Editorial: Vegetation, ecosystem processing and carbon budget of wetlands under global change

**DOI:** 10.3389/fpls.2023.1160319

**Published:** 2023-03-30

**Authors:** Yong Li, Bing Song, Xiaoming Kang

**Affiliations:** ^1^ Wetland Research Center, Institute of Ecological Conservation and Restoration, Chinese Academy of Forestry, Beijing, China; ^2^ Beijing Key Laboratory of Wetland Services and Restoration, Beijing, China; ^3^ Sichuan Zoige Wetland Ecosystem Research Station, Tibetan Autonomous Prefecture of Aba, Sichuan, China; ^4^ School of Resources and Environmental Engineering, Ludong University, Yantai, China

**Keywords:** wetland, plant community, vegetation, ecosystem process and function, carbon cycling and sequestration, global change, carbon budget

The objective of this Research Topic is to synthesize how the components and functions of wetland respond to global change and gain novel insights into the underlying mechanisms. To answer this question, the Research Topic presents a collection of wetland research studies from different biomes to demonstrate how plant population and community, soil microbes, carbon recycling and carbon budget respond to changing environments and human activities.

The papers in this collection explored the coexistence mechanism of plant species in freshwater inland marshes and coastal wetlands. For example, the widespread species *Phragmites australis* had a higher competitive ability during the flooding period, while the endemic species *Triarrhena lutarioriparia* increased the dominance during the non-flooding period in the Dongting Lake wetlands with seasonal flooding, implying the niche differences between the two species enabled their coexistence (Du et al.). Moreover, dominant shrub species *Tamarix chinensis* and *Ziziphus jujuba* showed different water uptake sources during dry and wet seasons in the Yellow River Delta. The difference in interspecific water use strategies demonstrated these species have adapted to the fluctuations of soil moisture, contributing to successful coexistence and increasing the resilience of the coastal wetland ecosystem to drought (Zhu et al.). Furthermore, allelopathy is an important ecological adaptation mechanism for *P. australis* to maintain a high interspecific competitive advantage in the coastal wetlands, as observed in the Yellow River Delta in *P. australis* growing with *Suaeda salsa* (Gao et al.). These findings filled the gap in plant coexistence mechanism in wetlands, which must be important to understand the response of plant species under changing environments.

Responses of seed germinability, root morphological and chemical traits, carbon:nitrogen:phosphorus stoichiometry and plant diversity to changing environments and plant invasion were also investigated (Dou et al.; Feng et al.; Sun et al.; Xiong et al.; Zhao et al.). Seed germinability and viability of seven plant species in response to 90-day storage treatment including different groundwater level and salinity in supratidal wetlands of the Yellow River Delta were explored. It was found that once dispersed into habitats with high groundwater levels and high groundwater salinity in supratidal wetlands, many species of seeds might not germinate but maintain viability, highlighting the adaptation mechanism of seed to environmental changes (Feng et al.). *T. chinensis* responded to the heterogeneity of soil water and salinity induced by groundwater level through trade-offs and changes in root morphological, nutrients and chemical characteristics in coastal wetland of the Yellow River Delta (Sun et al.). The homeostasis results of C, N and P elements in three halophytes (salt-secreting *Aeluropus sinensis*, salt-repellent *Phragmites. communis* and salt-accumulating *S. salsa*) showed that the homeostasis was strongest in *A. sinensis* and weakest in *S. salsa* in the Yellow River Delta (Zhao et al.). These results might be attributable to the differences in plant C, N, P stoichiometry and salt accumulation. Xiong et al. examined the responses of plant-soil stoichiometry and plant growth strategy to land use change (mudflat, native *P. australis*-dominated wetland, invasive *Spartina alterniflora*-dominated wetland, and reclaimed *P. australis*-dominated wetland) in Hangzhou Bay coastal wetland. Land use exerts a strong effect on plant organ and soil depth C:N:P stoichiometry and plant growth strategies. Furthermore, the effects of increased freshwater inputs were explored in coastal wetlands of Yancheng and demonstrated strong benefits in the control of invasive species such as *S. alterniflora*, modifying interspecific plant relationships and increasing plant diversity (Dou et al.). These findings provide insights into responses of plant growth strategies to changing environments.

Beside plants, responses of microbial communities and microbial diversity to wetland degradation and climate warming were also explored (Abulaizi et al.; Li et al.; Wang et al.). Li et al. investigated the diversity of nitrogen-fixing bacteria at different stages of degradation characterized by number and size of freeze-thaw mounds in the alpine wetland of Qinghai-Tibet Plateau. The results showed that the degradation of alpine wetland inhibited the growth of nitrogen-fixing bacteria, leading to the decline of their nitrogen-fixing function. Abulaizi et al. found that the vegetation communities and soil nutrients changed significantly with increasing soil degradation levels, and *Sphingomonas* could be used as a potential biomarker of degradation in mountain wetlands. Moreover, the vertical structure and assembly of the soil bacterial and fungal communities along the soil profile responded differently to short-term warming in Zoige alpine peatland. Results demonstrated that warming significantly influence the vertical structure of the fungal community but not bacterial community and that the vertical structure of the fungal community was driven by a dispersal-based process under control treatment, while the niche and dispersal processes jointly regulated the fungal communities under warming treatment (Wang et al.). These findings could provide insights into the underlying mechanism of microbial communities in response to changing climate and environment in wetlands.

How vegetation responds to climate change and human activities have been discussed in this Research Topic as well (Deng et al.; Wang et al.). Deng et al. found that human activity, rather than climate change was the main reason for vegetation improvement or degradation in the Jilin Momoge marshes of the semi-arid region of northeast China. Wang et al. explored the spatiotemporal change of aboveground biomass and its response to climate change in a marsh wetland of western Songen Plain using MODIS datasets from 2000 to 2020. The increase of summer and autumn precipitation stimulated aboveground biomass by promoting vegetation growth during 2000–2020. These results provide theoretical guidance for the restoration, protection, and adaptive management of wetland vegetation in changing environments.

This Research Topic also explored the responses of ecosystem functions such as nitrogen and phosphorus sinks, carbon recycling and carbon budget to changing environment (Peng et al.; Wang et al.; Zhu et al.). For example, Peng et al. explored the harvest effect on plant nitrogen and phosphorus sinks and these two elements release from litter decomposition of the dominant species *Miscanthus lutarioriparius* in the Dongting Lake wetlands. Non-harvest treatment greatly decreased nitrogen and phosphorus sinks, compared to the harvest treatment. Furthermore, Wang et al. found that Zoige alpine meadow in the Qinghai-Tibet Plateau acted as a faint carbon source during the 5-year observation periods and the annual net CO_2_ exchange in the alpine meadow ecosystem was mainly controlled by the maximum CO_2_ release rates. Zhu et al. studied the carbon budget of the growing season at long-term and its driving mechanism in a alpine wetland in the Qilian Mountains (northeastern Qinghai-Tibet Plateau) from 2004–2016. Climate warming could be beneficial to the enhancement of gross primary productivity and ecosystem respiration in the alpine wetland, while the increase of precipitation could weaken this effect. These results could advance our understanding of ecosystem function response to various environmental factors in the context of global change.

Overall, this Research Topic provides a synthesis of plant and microbial communities, vegetation, and ecosystem functions of wetlands (including inland lakes, freshwater marshes, mountain wetlands and coastal wetlands) in response to environmental changing because of human activities. We hope these findings could provide new insights into guidance for the conservation and restoration of wetlands under global change.

## Author contributions

YL and BS wrote the first version of manuscript. XK revised the manuscript and all the authors contributed to the final version of the manuscript.

